# Percutaneous mechanical support in catheter ablation of ventricular arrhythmias: hype or hope?

**DOI:** 10.1093/europace/euae186

**Published:** 2024-07-19

**Authors:** Josef Kautzner, Jana Hašková, Predrag Stojadinovič, Petr Peichl, Dan Wichterle

**Affiliations:** Department of Cardiology, Institute for Clinical and Experimental Medicine, Vídeňská 1958/9, Prague 140 21, Czech Republic; Department of Internal Medicine I – Cardiology, Palacký University Medical School, Olomouc, Czech Republic; Department of Cardiology, Institute for Clinical and Experimental Medicine, Vídeňská 1958/9, Prague 140 21, Czech Republic; Department of Internal Medicine I – Cardiology, Palacký University Medical School, Olomouc, Czech Republic; Department of Cardiology, Institute for Clinical and Experimental Medicine, Vídeňská 1958/9, Prague 140 21, Czech Republic; Department of Cardiology, Institute for Clinical and Experimental Medicine, Vídeňská 1958/9, Prague 140 21, Czech Republic; Department of Cardiology, Institute for Clinical and Experimental Medicine, Vídeňská 1958/9, Prague 140 21, Czech Republic

**Keywords:** Catheter ablation, Ventricular tachycardia, Substrate modification, Percutaneous mechanical support, Stereotactic arrhythmia radiotherapy

## Abstract

Catheter ablation (CA) has become an established treatment strategy for managing recurrent ventricular tachycardias (VTs) in patients with structural heart disease. In recent years, percutaneous mechanical circulatory support (PMCS) devices have been increasingly used intra-operatively to improve the ablation outcome. One indication would be rescue therapy for patients who develop haemodynamic deterioration during the ablation. However, more efforts are focused on identifying subjects who are at high risk of such deterioration and could benefit from the pre-emptive use of the PMCS. The third reason to use PMCS could be the inability to identify diffuse substrate, especially in non-ischaemic cardiomyopathy. This paper reviews available experiences using various types of PMCS in different clinical scenarios. Although PMCS allows mapping during VT, it does not significantly influence acute outcomes and not convincingly long-term outcomes. On the contrary, the complication rate appears to be higher in PMCS cohorts. Our data suggest that even in patients with severe left ventricular dysfunction, the substrate modification can be performed without the need for general anaesthesia and risk of haemodynamic decompensation. In end-stage heart failure associated with the electrical storm, implantation of a left ventricular assist device (or PMCS with a transition to the left ventricular assist device) might be the preferred strategy before CA. In high-risk patients who are not potential candidates for these treatment options, radiotherapy could be considered as a bail-out treatment of recurrent VTs. These approaches should be studied in prospective trials.

## Graphical Abstract

**Graphical Abstract:**
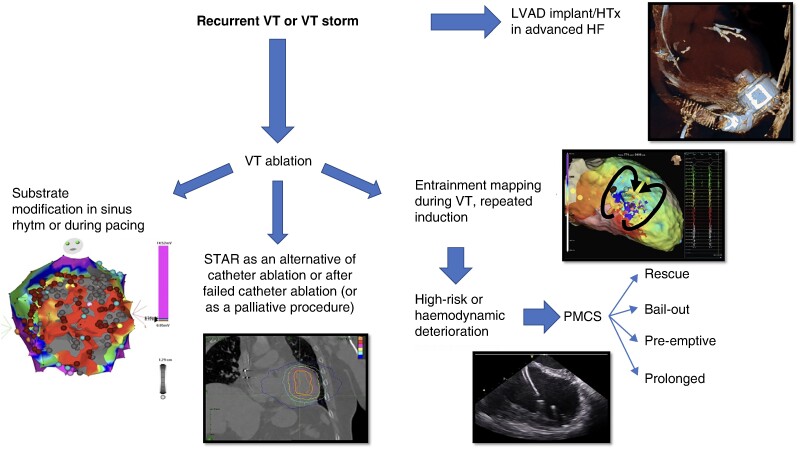
HTx, heart transplant; LVAD, left ventricular assist device; PMCS, percutaneous mechanical cardiac support; STAR, stereotactic arrhythmia radiotherapy; VT, ventricular tachycardia.

**What’s new?**

- Percutaneous mechanical circulatory support (PMCS) has been increasingly used to support catheter ablation for ventricular tachycardias (VTs).
- Pre-emptive use of PMCS in a high-risk population may help to improve the acute results of ablation; however, no clear-cut data supporting long-term benefits are available.
- PMCS may be helpful to enable mapping in VT among patients with less defined myocardial substrates.
- Our preliminary analysis of a large cohort of patients undergoing ablation of VT suggests that substrate-based ablation may be associated with a low risk of haemodynamic deterioration.
- The unresolved question is whether to perform ablation on PMCS in haemodynamically compromised patients with severe heart failure or whether to implant a preferentially left ventricular assist device (or PMCS with a transition to left ventricular assist device) instead of catheter ablation.
- In high-risk patients who are not potential candidates for these treatment options, stereotactic arrhythmia radiotherapy could be considered as a bail-out therapy for recurrent VTs.

## Introduction

Catheter ablation (CA) has become an established treatment strategy for managing recurrent ventricular tachycardias (VTs) in patients with structural heart disease. Ablation decreases arrhythmia burden and implantable cardioverter-defibrillator (ICD) therapies, improves the quality of life, and reduces the need for antiarrhythmic drugs. Repeated CA may be necessary to achieve this goal. Furthermore, CA is considered a lifesaving procedure in intractable VTs or in electrical storm.^1–4^

Since VTs are often unmappable due to haemodynamic instability or inability to induce in the electrophysiology laboratory, contemporary ablation strategies are focused mainly on substrate mapping and modification. Even this strategy may be complicated by triggering multiple VTs requiring repeated cardioversions or haemodynamic deterioration. In addition, many operators still prefer to use at least limited high-density mapping or entrainment mapping during VT. Multiple episodes of VT may decrease organ perfusion, lactic acidosis, catecholamine release, and activation of inflammatory cytokines. The consequences could be acute intra-procedural instability or post-procedural decompensated heart failure. Such deterioration is associated with an increased mortality.

In recent years, percutaneous mechanical circulatory support (PMCS) devices have been increasingly used intra-operatively to improve the outcome of CA.^5^ The brief summary of available technologies is presented in *Table 1*. One indication for PMCS would be rescue therapy for patients who develop haemodynamic deterioration during the ablation, which is non-responsive to catecholamines. However, more efforts are focused on the identification of subjects who are at high risk of such deterioration and could benefit from the pre-emptive use of the PMCS. The third reason to use PMCS could be the inability to identify diffuse substrate, especially in non-ischaemic cardiomyopathy.

**Table 1:** Available PMCS systems

Device	Access	Mechanism	Level of support	Advantages	Limitations	Contraindications	Major complications
IABP	Percutaneous or surgical 7.5–8F	Counterpulsation (systolic unloading, diastolic augmentation)	0.5 L/min	Know-how Ease of use Small diameter for access	Modest augmentation of CO Uses ECG or BP triggers, not optimal for VT	Moderate to severe aortic regurgitation Aortic valve disease Severe PAD	Limb ischaemia Renal or gut ischaemia Vascular injury Stroke
TandemHeart	Percutaneous or surgical 21F inflow (transseptal) 15 or 17 F outflow	Centrifugal continuous flow pump	3.5–5.0 L/min	Complete LV support	Large cannulas, requires TS access	Severe PAD Ventricular septal defect Severe RV failure	Limb ischaemia Vascular injury Tamponade Stroke Vascular injury Residual ASD Air embolism
Impella 2.5 Impella CP Impella 5.0	Percutaneous or surgical Single arterial access 13F Percutaneous or surgical Single arterial access 14F Surgical cutdown Single arterial access 21F	Axial flow pump (pumping blood from LV to aorta)	2.5 L/min 3.5 L/min 5.0 L/min	Partial LV support Partial LV support Complete LV support	Large arterial cannula Large arterial cannula Large arterial cannula	Mechanical aortic valve Aortic stenosis Aortic valve disease Moderate to severe aortic regurgitation Severe PAD LV thrombus Ventricular septal defect RV failure	Limb ischaemia Vascular injury Perforation Stroke Haemolysis Limb ischaemia Vascular injury Perforation Stroke Haemolysis Limb ischaemia Vascular injury Perforation Stroke Haemolysis
VA ECMO	Percutaneous or surgical 17–22F venous, 15F arterial	Centrifugal continuous flow pump with oxygenator	>4.5 L/min	Highest level of LV support Usable in RV failure	Large cannulas Complex setup	Severe PAD Uncontrollable coagulopathy	Limb ischaemia Vascular injury Pulmonary oedema Bleeding sepsis Thrombus Systemic embolism

ASD, atrial septal defect; BP, blood pressure; CO, cardiac output; IABP, intra-aortic balloon pump; LV, left ventricular; PAD, peripheral artery disease; PMCS, percutaneous mechanical cardiac support; RV, right ventricular; VA ECMO, venoarterial extracorporeal membrane oxygenation; VT, ventricular tachycardia.

This review attempts to summarize the available evidence on the clinical utility of PMCS to support VT ablation in structural heart disease. We will also present our somewhat controversial view of the problem, which is based on long-term experience with CA of VTs in a high-volume centre. Finally, we will touch on the possible role of radiotherapy in managing high-risk patients with recurrent VTs. Important clinical studies on this topic are listed in *Table 2* for easier orientation.

**Table 2:** Summary of PMCS studies in VT ablation

Author	Device	Design	No of patients	Age (years)	LVEF	CAD (%)	FU	Main results
Zanobini *et al*.^6^	Centrifugal pump w. oxygenator	Retrospective observational	8	69.5 ± 5	38 ± 16	7/8	6 months	Successful ablation of 15/21 VTs, all patients free of recurrences
Carbucicchio *et al*.^7^	ECMO	Retrospective observational	19	61 ± 6	28	58	42 months	Inducible 62 VTs, 56/62 required support Non-inducibility at the end 53% At 6 months, 28% VT-free
Miller *et al*.^8^	Impella 2.5	Retrospective observational	10 vs. 13 (non-PMCS)	64.1 ± 13.4 and 66.8 ± 16	26 ± 11 vs. 35 ± 18	60 vs. 39	3 months	34 VTs in 10 patients, 38 VTs in 13 (21 VTs terminated during vT), no difference between groups in inducibility at the end (25 vs. 33%) During FU, 30 and 31% patients had VT recurrences
Bunch *et al*.^9^	TandemHeart	Retrospective observational	13 vs. 18 (non-PMCS)	59.7 vs. 62.5	20 vs. 25	61.5 vs. 67.7	9 ± 3 months	Acute procedural success in vs. 67%, freedom from clinical VT 83.3 vs. 61.1 (n.s.)
Miller *et al*.^10^	Impella 2.5	Prospective interventional	20	58.5 ± 11.6	28.9 ± 6.5	35	30 days	VT terminated during RF ablation in 67%, 75% non-inducible at the end, death in 10%, hospitalization in 25%, recurrence of sustained VT 20%
Lu *et al*.^11^	ECMO vs. Impella 2.5 vs. HeartMate	Prospective interventional	16 (5, 5, 6)	63 ± 11	20 ± 9	87	3 months	77 VTs induced, clinical VT terminated in all, acute success in 60,60 and 50%, VT inducible in 44% at the end Clinical success over 3 months—reduction of median from 6 to 0.2 VT episodes
Aryana *et al*.^12^	Impella 2.5/CP	Retrospective observational	68 (34 PMCS, 34 control)	66 ± 12	32 ± 10	53	19 ± 12 months	Procedural success 71% in both groups, recurrences in 26% and 41% (n.s.), composite endpoint (30 day reshospitalization, re-do ablation, ICD therapies and 3-month mortality lower in PMCS (12 vs. 35, *P* < 0.04),
Baratto *et al*.^13^	ECMO	Retrospective observational	64	62 ± 15	37 ± 14	45	23 ± 13 months	VT non-inducibility in 69%, VT recurrence in 33%, overall survival 88% Non-inducibility correlated with all-cause death, LVAD and heart transplantation
Turagam *et al*.^14^	ECMO/Impella 2.5/TandemHeart	Retrospective observational	1655 (105 on PMCS)	63.6 ± 11.2	25.4 ± 11.9	53	527 days	Acute procedural success lower in PMCS group (71.8 vs. 73.7%, *P* = 0.04) and 1 year mortality higher (34.7 vs. 9.3%, *P* < 0.001). Matched groups by EF and NYHA III–IV showed no difference in outcomes.
Mathuria *et al*.^15^	Impella/TandemHeart	Observational	93 (12 rescue PMCS, 24 pre-emptive, 57 control)	66 ± 6	26.6 ± 9	55	3 months	30 days mortality was higher in the rescue group compared with the pre-emptive group (58 vs. 4%, *P* = 0.003) and control (58 vs. 3%, *P* < 0.001)
Kusa *et al*.^16^	Impella 2.5/CP	Retrospective, observational	194 (109 PMCS, 85 control)	64 ± 11	26 ± 10	56	215 days	VT inducibility (20 vs. 7%; *P* = 0.02), and length of subsequent hospitalization (median 6 vs. 4 days; *P* = 0.001) higher in PMCS group. Primary endpoint (recurrent VT, transplant or death) occurred in 36 vs. 26% (ns)
Muser *et al*.^17^	Impella 2.5/CP	Retrospective, propensity matched	150 (75 in each group)	65 ± 13	26 ± 11	53	9 months	Peri-procedural AHD occurred in 5 (7%) patients prophylactic PMCS and in 17 (23%) patients in the control population (*P* < 0.01). The 12-month cumulative incidence of VT was 40% in the prophylactic PMCS group vs. 41% in the control group (*P* = 0.97).
Enriquez *et al*.^18^	ECMO rescue	Observational	21 storm patients	61 ± 11	21.1 ± 13.2	90	10 days	VF suppression in 89%, VT non-inducibility in 78%. A total of 16 patients died and 7 survived (5 free of VT/VF).
Di Monaco *et al*.^19^	ECMO	Observational	19	62 ± 10	28.2 ± 10.4	79	10 months	VT non-inducible in 16/19 (84%), during FU, 3 patients died from CHF and 1 from VT/VF. Overall procedural success rate 64%.
Neuzner *et al*.^20^	Impella 2.5/CP	Retrospective observational	26 (pre-emptive PMCS in 25)	68 ± 9	19.6 ± 3	80	N/A	Ablation performed in all 25 with pre-emptive PMCS, not in 1 with rescue PMCS. All inducible VTs ablated.
Ballout *et al*.^21^	IABP in14, Impella CP in 2, ECMO in 1, combination in 3	Prospective registry on bail-out ablation	21	61 median	20 median	81	N/A	VT non-inducible in 19 (91%), 81% weaned from PMCS, 29% died because of shock
Grimaldi *et al*.^22^	ECMO	Observational	62 (31 ECMO, 31 no ECMO)	68 ± 9	30 ± 9	76	median 24 months	Four patients in control (13%) and five patients in PMCS (16%) died due to refractory heart failure. An ICD intervention (shock or anti-tachycardia pacing) was documented in 13 patients of control group (42%) and 6 patients in PMCS (19%).
Chen *et al*.^23^	Impella 2.5 or CP	Retrospective observational	61 (43 early, 26 delayed removal of PMCS)	65.6 ± 9.3 vs. 64.6 ± 13.8	27.1 ± 9.3 vs. 20. 6 ± 5.4 (*P* < 0.002)	79.1 vs. 65.4	90 days	At 90-day follow-up after VT ablation, 14/69 (20.3%) patients died. Mortality rate was higher in delayed removal group (50 vs. 2.3%, *P* < 0.001). Delayed removal was only predictor of 90-day mortality.

AHD, acute haemodynamic decompensation; CAD, coronary artery disease; CHF, chronic heart failure, ECMO, extracoporeal membrane oxygenation, FU, follow-up; ICD, implantable cardioverter-defibrillator; LVAD, left ventricular assist device; LVEF, left ventricular ejection fraction; NYHA, New York Heart Association; PMCS, percutaneous mechanical circulatory support; RF, radiofrequency, VF, ventricular fibrillation; VT, ventricular tachycardia.

## What did the early studies reveal?

The first report on using PMCS was published by Zanobini *et al.* in 2003.^6^ The authors reported on the outcome of eight patients who underwent CA of haemodynamically untolerated VT. Percutaneous mechanical circulatory support (centrifugal pump with oxygenator, an imperfect predecessor to venoarterial extracorporeal membrane oxygenation (VA ECMO) was used on average for 140 min and allowed mapping of VTs, maintaining the mean arterial pressure of 65 mmHg. Notably, the active support use was only intermittent. Its duration was limited to 15- to 20-min intervals, after which the tachycardia was interrupted. Successful ablation of 15 of 21 VTs was achieved (71%). Four patients had no inducible VTs during the procedure, and no significant complications were observed. All patients were free of VT episodes in the next 6 months. This study suggested a reasonable approach of intermittent PMSC for relatively short episodes of VT, which was not surprisingly used in subsequent studies.

Carbucicchio *et al.*^7^ also used the same type of imperfect PMCS to support emergent CA in 19 patients with acute haemodynamic decompensation or electrical storm. Catheter ablation was guided predominantly by activation mapping and abolished 45/56 of VTs. All clinical VTs were suppressed in 10/19 subjects. The mean procedural time was 4 h and 20 min. Complete stabilization was achieved in 13 patients (68%) without recurrence during 7 days hospital monitoring. At a mean follow-up of 42 months (range 15–60 months), 5/18 patients (28%) were free from VT recurrence, 7/18 (39%) had a clear clinical improvement with reduced ICD interventions, 5/14 patients (36%) had a recurrence of the storm, and three of them died because of acute heart failure. No PMCS-related serious complications were observed.

The Mayo Clinic team published the first US case report on using PMCS during VT ablation.^24^ They used TandemHeart MCS in a patient with dilated cardiomyopathy and recurrences of VT despite massive antiarrhythmic therapy and previous substrate-based ablation. After installation of PMCS, induced VT was haemodynamically tolerated for 1 h and 45 min, allowing endocardial mapping. The subsequent drop in blood pressure necessitated cardioversion, and CA was performed epicardially opposite the endocardial linear lesion that rendered VT non-inducible. Post-procedural weaning from PMCS was fast. Notably, the patient remained for the next 7 months without recurrences of VT. This case showed again that effective PMCS allows the mapping of otherwise unmappable VT.

Another early observational study on the use of PMCS in the ablation of unstable VT was published by Miller *et al*.^8^ The study included 10 ablations on the Impella 2.5 PMCS device, and 13 other ablations were performed using an intra-aortic balloon pump (6 procedures) or without device support (7 procedures). The main result was that in patients on Impella, VT was tolerated and mapped for a significantly more extended period, and more VTs were terminated during CA. Using continuous haemodynamic monitoring with cerebral oximetry to monitor adequate cerebral perfusion, they found no difference in significant drops in cerebral oximetry between the PMCS cohort and the non-supported patients. However, patients in the PMCS-supported group were maintained in VT for longer. There was no difference in VT recurrences during the follow-up. In another study, the same group confirmed a more favourable haemodynamic profile compared with inotropes during fast simulated VT and emphasized the role of cerebral oximetry for safety monitoring.^10^

The US experience with different types of mechanical support for VT ablation in 16 patients with unstable VT was presented by Lu *et al*.^11^ The support consisted of Impella 2.5 in five, an implantable left ventricular assist device (LVAD) in six, and a peripheral cardiopulmonary bypass in five. Except for two Impella cases, haemodynamic support was adequate to permit sufficient activation mapping for CA. Time in VT was 78 ± 36 min. Three complications occurred in the Impella group. The median number of ICD therapies decreased from six in the month before ablation to zero in the following month.

In summary, the first studies showed the feasibility of PMCS use in ablating unstable VTs. Studies with centrifugal pump with oxygenator and intermittent mapping during VT demonstrated safety and reasonable efficacy. Also, mapping during VT for a longer time using different types of PMCS was demonstrated to be feasible. However, some complications occurred, and there was no evidence that this approach improved the long-term results of CA.

## Real-life data?

The first attempt to describe multicentre experience with PMCS was published by Aryana *et al*.^12^ They analysed a cohort of 68 unstable patients who underwent mapping and ablation of scar-related VT using PMCS (*n* = 34) vs. no support (*n* = 34). Even though more VTs were terminated in the PMCS group, the procedural success rate was identical (71%). Recurrences were similar during the follow-up. However, the composite endpoint of 30-day rehospitalization, re-do VT ablation, ICD therapies, and 3 months mortality was lower with PMCS (12%) than without (35%). Larger retrospective study investigated the VT ablation outcomes in the Medicare Inpatient Standard Analytic File database, containing 345 patients (PMCS introduced in 230, intra-aortic balloon pump in 115).^25^ It showed that compared with VT mapping and ablation on intra-aortic balloon pump, PMCS-assisted procedure was associated with reduced in-hospital cardiogenic shock, renal failure, length of stay, hospital re-admissions, and mortality. Interestingly, no difference in re-do VT ablation was observed at 1 year of follow-up.

Another evidence reflecting everyday clinical practice at the Mount Sinai Hospital was a study by Kusa *et al*.^16^ The study population comprised 194 patients (109 PMCS and 85 non-MCS). The PMCS group patients were sicker, and they had higher New York Heart Association (NYHA) heart failure class III (51 vs. 25%; *P* < 0.001), lower left ventricular ejection fraction (LVEF) (26 ± 10 vs. 39 ± 16%; *P* < 0.001), and more often were in an electrical storm (49 vs. 34%; *P* = 0.04). Procedure times (422 ± 112 vs. 330 ± 92 min; *P* < 0.001), post-ablation VT inducibility (20 vs. 7%; *P* = 0.02), and length of subsequent hospitalization (median 6 vs. 4 days; *P* = 0.001) were all higher in the PMCS group. During the median follow-up of 215 days, recurrent VT, heart transplantation, or death occurred similarly in both groups (36% in PMCS vs. 26% in non-MCS groups; *P* = 0.14). After propensity matching for differences between both groups, the procedural outcomes and endpoints were not different. This neutral result was explained as the lack of statistical power or as an interaction with other confounders. Importantly, the study did not compare substrate-based ablation with PMCS but rather compared mapping during VT on intra-aortic balloon pump with mapping on advanced PMCS.

In summary, studies using PMCS as a part of the routine workflow in procedures that were considered high-risk suggested that there might be some benefit in very selected groups of patients. Again, no apparent advantage in long-term outcomes was observed. Data from Medicare suggested some advantages of PMCS for better in-hospital outcome. However, ablation on PMCS was compared with a group of patients where CA was based primarily on activation mapping without PMCS. This fact, together with the use of general anaesthesia, could have resulted in the deterioration of the clinical status of patients ablated without PMCS.

## Rescue mechanical support

The use of PMCS when haemodynamic deterioration occurs during ablation of VT or ventricular fibrillation (VF) is called rescue PMCS. In a study by Enriquez *et al.*,^18^ from the UPenn group, a total of 21 patients with an electrical storm (11 due VT, 10 due to focally triggered VF) presented with peri-procedural decompensation, requiring ECMO support. In 14 patients, decompensation occurred before and in the remaining 7 during or after the procedure. Ablation was performed in 18 patients (9 with VF and 9 with VT). In patients with VF, suppression of triggering ventricular ectopy was achieved in 8/9 (89%) cases. In those with VT, non-inducibility was achieved in 7/9 (78%). After a median follow-up of 10 days, 16 patients died (13 during the index admission). Death was due to refractory VT/VF in 4 cases, heart failure in 11, and non-cardiac cause in 1 case. Seven patients survived beyond 6 months post-ablation; five remained free of VT/VF, and three ultimately received destination therapy (heart transplantation in two and LVAD in one). Complications included significant bleeding in three subjects from vascular access sites, thrombosis of ECMO cannula with compromise in oxygenation, and one left ventricular thrombus with embolization.

In summary, studies like this may suggest that the rescue use of PMCS has relatively poor results. However, the outcome reflects, to a great extent, the underlying cause of the storm. Half of the patients in the above study presented with VF; such CA could be tough without a PMCS. This scenario will remain a good indication for PMCS of ECMO type and may also enable patient transportation to the ablation centre. However, in patients with monomorphic VTs and severe heart failure, one should consider between pragmatic substrate modification without general anaesthesia and repeated programmed stimulation or implantation of the LVAD.

## Bail-out ablation on mechanical support

Ballout *et al*.^21^ reported on 21 consecutive patients with cardiogenic shock and concomitant refractory ventricular arrhythmia undergoing bail-out ablation due to the inability to wean off mechanical support. This is a different category compared with the rescue use of PMCS where support is provided due to haemodynamic deterioration during CA. The median LVEF in this cohort was 20%, and 81% had ischaemic cardiomyopathy. Prevailing PMCS was an intra-aortic balloon pump (67%), less frequently Impella CP or ECMO. Endocardial mapping showed scars in 90% of patients, and VT was inducible in 13 of them (62%), 6 patients had VF triggered by premature beats. Activation mapping was possible in all 13 inducible patients, and substrate modification was performed in 15 patients with scar. Seventeen subjects were weaned of the PMCS (81%), and six died due to persistent cardiogenic shock. Patients who had ventricular arrhythmia and cardiogenic shock on presentation had a trend towards lower in-hospital mortality compared with those who presented with cardiogenic shock and later developed ventricular arrhythmia.

In summary, CA on PMCS may be performed as a bail-out procedure in subjects who cannot be weaned from previously implanted PMCS. Since these patients do have often advanced heart failure, their prognosis remains poor.

## Pre-emptive use of mechanical support

The idea of pre-emptive implantation of PMCS prompted a search for predictors of haemodynamic deterioration in VT ablation. Recently, the UPenn group has proposed a risk score (PAINESD) incorporating clinical and procedural variables that may help identify patients at high risk of peri-procedural acute haemodynamic decompensation who may benefit from prophylactic use of PMCS.^26^ The same group tested this score in a clinical study, including 75 high-risk patients with ablation on PMCS and 75 propensity-matched subjects who did not undergo prophylactic MCS placement.^17^ Prophylactic PMCS placement in high-risk patients undergoing CA of scar-related VT was associated with a reduced risk of acute haemodynamic decompensation and death/transplant during follow-up without impacting VT-free survival.

Based on PAINESD score, Neuzner *et al*.^20^ used pre-emptively PMCS (Impella 2.5) for ablation of VT in 25 patients with severe left ventricular dysfunction. In another patient, PMCS was used as a rescue therapy. All inducible VTs were activation mapped and ablated. Acute decompensation occurred only in one patient with rescue PMCS.

A different approach to risk stratification was introduced in a larger study on the predominantly pre-emptive use of PMCS to support CA published by Baratto *et al*.^13^ Arrhythmia pattern, haemodynamic state, left ventricular systolic function, extent of coronary artery disease, and the presence of comorbidities (chronic kidney disease defined as serum creatinine ≥ 1.5 mg/dL and severe pulmonary disease based on the presence of PCO_2_ > 50 mmHg) were used to classify patients into a high- and low-risk group. A total of 74 procedures with ECMO support were performed in 64 out of 781 consecutive patients admitted within 5-year period. VA ECMO was used as a pre-emptive strategy in 59/64 patients (92%). Interestingly, 22% of the cohort had a failure of previous substrate-based procedures with persistent induction of unmappable VT (five of them in the low-risk cohort). Ablation tactics consisted of activation mapping complemented with substrate modification. Ventricular tachycardia was terminated in 81% of procedures with baseline inducible VT, and VT non-inducibility was achieved in 69%. Acute heart failure occurred in five patients: three underwent emergency heart transplantation, one had LVAD, and one patient eventually died. After a median follow-up of 21 months (13–28 months), VT recurrence was 33%; overall survival was 56 out of 64 patients (88%). Ventricular tachycardia recurrence was related to ablation success (after 180 days of follow-up: 19% when VT was non-inducible, 42% if non-clinical VT was inducible, 75% when clinical VT was inducible, and 75% in untested patients; *P* < 0.001). At multivariable analyses, non-inducibility (hazard ratio 0.198; *P* = 0.001) and LVEF (hazard ratio 0.916; *P* = 0.008) correlated with all-cause death, LVAD, and heart transplantation.

The pre-emptive use of ECMO for haemodynamically unstable VTs was also reported by Di Monaco *et al*.^19^ Catheter ablation in the setting of an electrical storm was performed in 19 patients, enabling activation mapping in all of them. Ventricular tachycardias were not inducible after CA in 16/19 patients (84%). Two patients underwent femoral artery stenting for dissection, and one had haemorrhagic shock due to the dislodgement of the arterial cannula. During the 10-month follow-up, three patients died of refractory heart failure and one due to an electrical storm.

A comparison of rescue use of PMCS with pre-emptive insertion and no haemodynamic support in 93 patient cohort was published by Mathuria *et al*.^15^ Thirty-day mortality was higher in the rescue group as compared with the pre-emptive group (58 vs. 4%; *P* = 0.003) or non-PMCS group (58 vs. 3%; *P* = 0.001). While there was a difference in PAINESD score between rescue PMCS and non-PMCS groups (17.8 vs. 13.4; *P* = 0.01), there was no difference in the PAINESD score between the pre-emptive and rescue PMCS cohort (17.8 vs. 16.5; *P* = 0.47).

In summary, the above studies show that the pre-emptive use of PMCS may be reasonably safe and allow the ablation of the most inducible VTs. However, these studies do not answer whether PMCS is really needed to support ablation in high-risk patients if pacemap-guided substrate ablation is used instead of a mapping strategy in VT. The fact that one cannot achieve the non-inducibility of several VTs in high-risk patients does not mean that their prognosis will be worse. It might be that the severity of heart failure predominantly determines the outcome, and VTs are more or less a marker of poor prognosis. Then, it comes again to a question whether patients with severe heart failure and acute decompensation during VT should not undergo implantation of LVAD rather than ablation on PMCS. The existing data suggest that this dilemma has to be solved on an individual basis.

## Comparison of activation mapping with substrate mapping

Interestingly, none of the above studies directly compared PMCS-assisted ablation with substrate mapping and ablation. Bunch *et al*.^9^ reported a case series of 13 consecutive patients with haemodynamically unstable VT who underwent a PMCS-assisted ablation and compared them with 18 disease-matched patients who underwent substrate modification in sinus rhythm without mapping during VT. Although the PMCS group showed a small benefit regarding a higher number of abolished clinical VTs or achievement of non-inducibility at the end of the procedure, long-term effects were identical. Even though the study claimed no statistical difference in the rate of complications between both groups, the actual number of severe complications appeared to be higher in the PMCS group (two strokes with one death, one tamponade, and one heart failure decompensation vs. one stroke, one ST elevation, and one decompensation of heart failure).

Grimaldi *et al*.^22^ compared two groups of patients with repeated episodes of haemodynamically unstable sustained ventricular arrhythmias. In the earlier period, ablations were performed without ECMO; later, ECMO was used pre-emptively. Substrate mapping was complemented by entrainment mapping during VTs. Without ECMO, all VTs were not mapped due to haemodynamic instability. At the end of substrate ablation, the programmed ventricular stimulation was not performed in 52% of patients due to haemodynamic instability. In the ECMO group, all inducible VTs were mapped and ablated. ECMO was removed at the end of the procedure in 30/31 patients. One patient required long-term support and died of refractory heart failure 5 days later. Five vascular complications were in the first group and three in the ECMO group. One patient had a haemorrhagic shock after the dislodgement of the ECMO cannula. During the median follow-up of 25 and 24 months, respectively, no difference in mortality was observed between both groups. An ICD intervention for sustained VT was more frequent in the first group (42 vs. 19%, *P* = 0.017). Interestingly, recurrences of VT in patients without inducibility at the end of the procedure were in a similar proportion of cases in both groups.

In summary, these studies suggested that even in high-risk patients, the long-term outcome of PMCS-supported ablation may be equivalent to substrate-based mapping and ablation.

## Multicentre experience

A total of 1655 patients from the International VT Ablation Center Collaborative group were analysed to describe the outcome with PMCS ablation, which was performed in 105 patients.^14^ Patients in the PMCS group had lower LVEF, higher NYHA functional class, and more ICD shocks, VT storm, and antiarrhythmic drug use (all *P* < 0.05). Both procedural and fluoroscopy times were longer in the PMCS cohort, and the acute procedural success was significantly lower with more complications and higher 1-year mortality. Multivariate Cox regression analysis demonstrated haemodynamic support as an independent predictor of mortality [hazard ratio: 5.01; 95% confidence interval (CI): 3.44–7.20; *P* < 0.001]. Subgroup analysis of patients with LVEF < 20% showed no difference in outcomes between both groups.

## Meta-analyses of available studies

More comprehensive data on the utility of PMCS emerge from a meta-analysis of studies comparing only the results of VT ablation on PMCS vs. no support. The first meta-analysis included five retrospective, observational studies of 2026 patients (MCS group—284 patients vs. non-MCS group—1742 patients).^27^ Similar to a study from Mount Sinai, the PMCS group was sicker with lower LVEF and a significantly higher proportion of VT storm and NYHA class ≥ III than non-MCS (*P* < 0.050). The acute procedural success (relative risk 0.95; 95% CI: 0.89–1.00; *P* = 0.070), VT recurrence (RR 0.94; 95% CI: 0.66–1.34; *P* = 0.740), and mortality (RR 1.28; 95% CI: 0.43–3.83; *P* = 0.660) were similar on follow-up for both groups. Both procedural and fluoroscopy times were more prolonged in the PMCS cohort (MD + 71.41 min, 95% CI: 31.67–111.14; *P* < 0.001 and MD + 7.31 min; 95% CI: 0.91–13.71; *P* = 0.030, respectively). Also, complications were significantly higher in the PMCS group (RR 1.83; 95% CI: 1.21–2.76; *P* = 0.004).

Another systematic review on the prophylactic use of PMCS included five observational studies presenting 400 procedures (PMCS: *n* = 187; no-MCS: *n* = 213).^28^ Meta-analysis compared the outcomes of both strategies. Two of these studies were analysed in the above meta-analysis; three were different. The primary outcome was in-hospital/30-day mortality. In the pro-MCS group, more VTs were induced (mean difference: 0.52, CI: 0.26–0.77; *P* < 0.0001), and patients remained in VT on average for 24.04 min longer (CI: 18.28–29.80; *P* < 0.00001). However, procedural success was comparable between groups, as was VT recurrence. In-hospital mortality was not different. An odds ratio for mortality at follow-up reached 0.55 (CI: 0.32–0.94; *P* = 0.03), suggesting that there may be some prognostic benefit. The most common PMCS-related complications were bleeding events.

A more recent meta-analysis included nine observational studies that compared clinical outcomes of VT ablation in patients with PMCS to controls without the support.^29^ The pooled data did not show a significant difference in mortality between both groups nor a difference in acute procedural success or in recurrence of VT. There was also no difference in the number of patients receiving cardiac transplant or being enrolled in the transplant list. Although there was no difference in the ablation time between groups, patients in the PMCS group had a longer total procedural time and more procedure-related adverse effects.

In summary, these meta-analyses confirm the initial observations. Although PMCS enables mapping during VT, it does not significantly influence acute outcome and not convincingly long-term outcomes. The complication rate appears to be higher in PMCS cohorts. Therefore, PMCS should be considered on an individual basis, especially in patients with severe electrical storms that do not seem to be triggered by terminal stage of heart failure. In the latter scenario, implantation of LVAD might be the better solution.

## Prolonged mechanical support

The authors studied a cohort of 69 patients who underwent VT ablation supported with an Impella 2.5 or CP pVAD (Abiomed, Danvers, MA, USA).^23^ The study population was divided into two groups, depending on the duration of the support. The early cohort (with PMCS removed within 24 h) was compared with a delayed cohort (with PMCS *in situ* up to 9 days). Mortality in the delayed cohort (50.0%) was significantly higher than in the early cohort (2.3%). Importantly, 12 out of 26 of patients in the delayed group were converted to other MCS post-procedurally.

In summary, this study seems to support the view that sicker patients with advanced heart failure and ventricular arrhythmias could be considered candidates for LVAD or heart transplant earlier instead of CA on PMCS.

## Can we predict haemodynamic deterioration?

**Figure 1:**
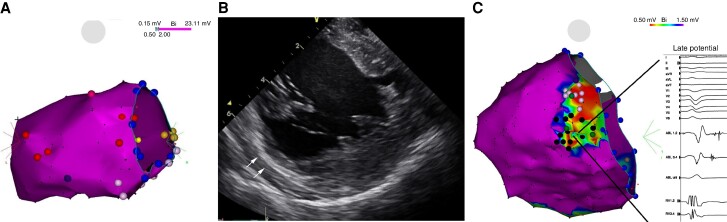
Electroanatomical voltage maps (CARTO 3, Biosense Webster) of the left ventricle in a patient with dilated cardiomyopathy and electrical storm. (*A*) Left panel shows an endocardial map with preserved voltage; (*B*) this panel displays intra-cardiac echocardiography with a clear mid-myocardial scar (arrows); and (*C*) this panel demonstrates an epicardial map with area of low voltage and late potentials in posterolateral region.

**Figure 2:**
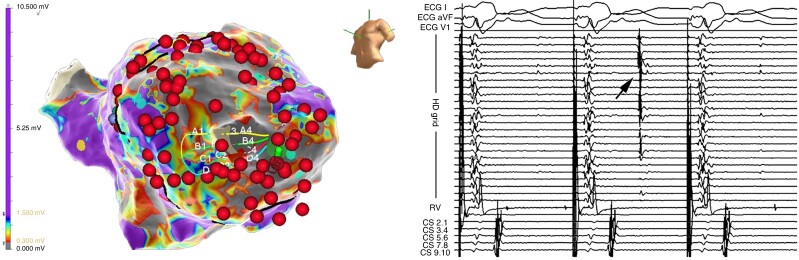
Electroanatomical voltage map of the left ventricle (Ensite Precision, Abbott) in a patient with anteroseptal aneurysm and electrical storm. Left panel shows lesion set that modified the substrate, achieved core isolation and non-inducibility of VT. Right panel displays endocardial signals from the multipolar mapping catheter (HD grid) and CS. Arrow marks the dissociation of the signal in the isolated region. ECG, electrocardiogram. CS, coronary sinus.

We recently analysed the data on the first VT ablation for structural heart disease-related VT between August 2006 and December 2020. The paper has been accepted to the *Europace* journal.^30^ The study cohort consisted of 1124 patients (age: 63 ± 13 years, males: 87%, ischaemic cardiomyopathy: 67%, electrical storm: 25%, NYHA class: 2.0 ± 1.0, LVEF: 34 ± 12%). Procedures were mainly performed under conscious sedation, which is very important for lower risk of haemodynamic deterioration. The only procedures under general anaesthesia (15% of all ablations) were cases of epicardial ablation or those where the patient was intubated due to an electrical storm before CA. Our mapping strategy is primarily based on substrate mapping using a combined approach (scar imaging with intra-cardiac echocardiography, voltage mapping, annotation of local abnormal ventricular activities and/or late potentials, pacemapping with an assessment of S-QRS intervals and QRS morphology at different sites with abnormal electrograms, and assessment of slowing conduction). Ablation strategy is always tailored to the individual substrate (*Figure 1*). In general, it focuses on eliminating abnormal or late potentials, interrupting conduction channels, and sometimes core isolation (*Figure 2*). In tolerated VT, entrainment mapping is used to confirm mapping data in sinus rhythm or during pacing. Programmed stimulation is done at the end of the procedure. In high-risk patients with severe left ventricular dysfunction, it may be even omitted. This is an important feature of our procedures—to avoid unnecessary inductions and mapping during VT unless well tolerated. The third unique feature of our study is the use of a purposely established institutional registry for complications of invasive procedures, which enables the collection of all peri-procedural complications that are subsequently adjudicated using the source medical records. Acute haemodynamic decompensation triggered by CA procedure was defined as intra-procedural or early post-procedural (<12 h) development of acute pulmonary oedema or refractory hypotension requiring urgent intervention. Despite the fact the PAINESD score in our large cohort was 11.4 ± 6.6 (median: 12, interquartile range: 6–17), acute haemodynamic decompensation occurred only in 13/1124 = 1.2% patients and was not predicted by the PAINESD score at all. Therefore, the PAINESD score showed no predictive value.

**Figure 3:**
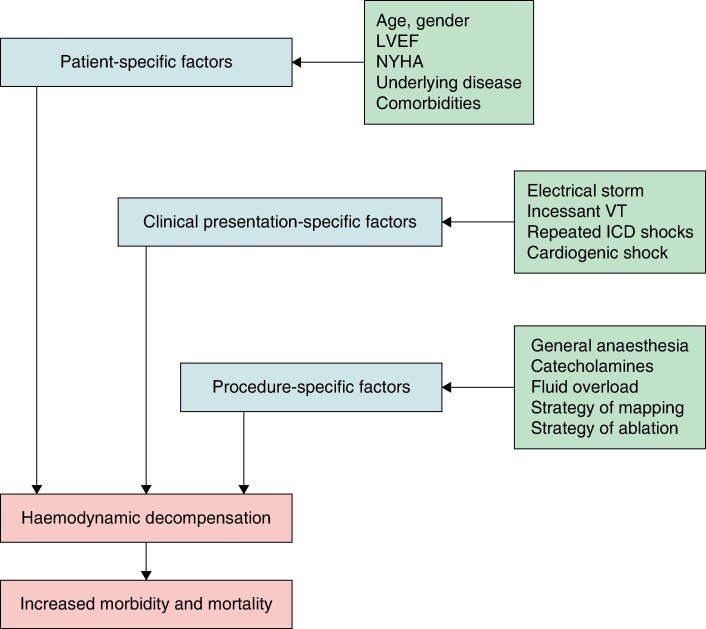
A scheme highlighting the main risk factors for haemodynamic decompensation during VT ablation. ICD, implantable cardioverter-defibrillator; LVEF, left ventricular ejection fraction; NYHA, New York Heart Association; VT, ventricular tachycardia.

**Figure 4:**
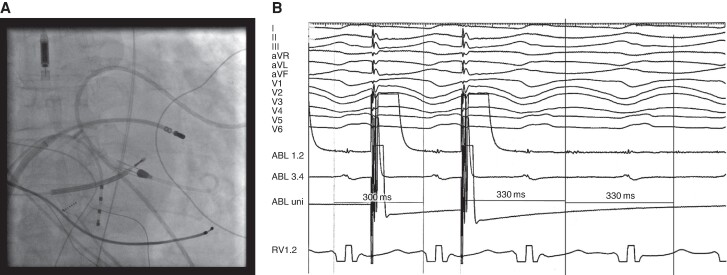
(*A*) Skiagram displaying deployed Impella 2.5 into the left ventricle and ablation catheter introduced transseptally into the central zone of re-entry circuit on the interventricular septum in a patient with dilated cardiomyopathy and recurrent unstable VTs. (*B*) Endocardial electrograms from ablation catheter (ABL) during entrainment pacing, showing mid-diastolic fragmented potentials and concealed entrainment. This site was not identified by substrate mapping in the sinus rhythm.

In general, the risk of haemodynamic deterioration during CA of VT in advanced heart failure has several components (*Figure 3*). They can be specified as follows: (i) patient-specific factors such as older age, ischaemic heart disease, severe LV dysfunction, and comorbidities (e.g. diabetes mellitus); (ii) factors related to the clinical presentation such as electrical storm or incessant VT; (iii) procedure-specific factors such as the strategy of CA (general anaesthesia, activation mapping vs. substrate mapping, the volume overload, repeated DC shocks). Since the first two groups of factors are the main components of the PAINESD score and the above analysis did not find the predictive value of this score, we believe that the key determinant of the risk is related to the strategy of the procedure. The crucial factors that can explain the lower risk of acute haemodynamic deterioration observed in our patient population include preference for conscious sedation over general anaesthesia, the use of substrate-based mapping as the dominant strategy of CA, intra-procedural haemodynamical monitoring (including monitoring of radiofrequency delivery and cardiac contractility with intra-cardiac echocardiography), and restrictive use of VT induction. In the case of severe left ventricular dysfunction, we even skipped programmed ventricular stimulation at the end of the procedure. This strategy did not seem to decrease efficacy of the procedure as estimated based on the number of re-ablations in our cohort.^23^ We used PMCS only in extremely rare cases of fast VT when the substrate was more diffuse and less identifiable during sinus rhythm or pacing and the patient was not in terminal stage of heart failure (*Figure 4*).

## Could radiotherapy solve the situation?

Stereotactic arrhythmia radiotherapy (STAR) has recently been suggested as a promising therapeutic alternative in cases of failed CA for recurrent VTs in patients with structural heart disease. Initial clinical experience with a single radiation dose of 25 Gy showed reasonable efficacy in reducing VT recurrences with acceptable acute toxicity and even with non-invasive mapping.^31,32^

Several case reports described successful rescue STAR in patients with intractable VT storm. The first report of successful STAR for VT storm was in a patient who was sedated and intubated.^33^ The treatment was non-invasive and well tolerated and showed an immediate benefit, avoiding CA. A study by Ninni *et al*.^34^ reported on a series of patients with recurrent electrical storm treated by STAR. The authors showed that STAR significantly reduced VT burden, but time to effectiveness varied from 1 to 7 weeks. Later recurrences were frequent but with longer cycle length in the majority of patients. Another report on the efficacy of STAR in a cohort of critically ill patients with advanced heart failure and recurrent VT documented an immediate reduction in VT burden.^35^ This acute reduction in VT burden helped to bridge five patients to heart transplant. However, late recurrences requiring ablation were observed in all patients surviving patients without transplant. Our recent study describing experience with STAR in patients with recurrent VT after previous CA confirms that STAR cannot be considered equivalent to CA but rather as a bail-out procedure after the failure of the previous CA.^36^

Another critical issue for discussion is the use of STAR in patients with LVAD and electrical storm. The experience is very limited since data on a small series of patients have been published.^37,38^ The results suggest that the procedure is able to prevent arrhythmia recurrence in some patients or reduce the VA burden. Importantly, all reported patients underwent at least one CA procedure in an expert centre, and all VAs were refractory to amiodarone. It is reassuring that no surgical problems were encountered in few patients who underwent subsequent heart transplant post-STAR.

In summary, these studies suggest that STAR could be considered in selected high-risk patients with a high threat of haemodynamic deterioration instead of considering CA supported by PMCS. Since it is still considered as a bail-out procedure, it could be indicated in subjects with severe comorbidities or with advanced heart failure without other treatment alternatives.

## Our view of the problem

Based on our 26-year experience with CA of VT/VF, we feel that most of cases, including electrical storms, can be safely ablated without general anaesthesia and using substrate modification without repeated induction of VT. As discussed above, this strategy results in low risk of acute haemodynamic decompensation with subsequent complications such as acute kidney injury. Such approach is supported by studies showing equivalent results of substrate-based and activation/entrainment-based ablation.^39^ When the patient has VT episodes or an electrical storm in the terminal stage of heart failure (Intermacs 1–3), we believe that it is more rational to implant ECMO with subsequent transition to LVAD rather than experiment with CA on PMCS. Similar strategies have been used by others.^40–43^

Even though there are insufficient data regarding such an approach, it appears to be a more reasonable tactic to stabilize patients and provide a safe bridge to invasive target treatment or heart transplant for the sickest patients with an electrical storm. This strategy can be argued against by the observation of frequent electrical storms after LVAD implant. Indeed, several studies reported the rate of electrical storm or recurrent VT in 7–21% of subjects.^44–49^ Some of them reported on reduced long-term survival, others on increased 30 day mortality. The pathophysiology of electrical storm after LVAD implant is not clear. Most studies agree on the fact that VAs are predominantly re-entrant, and a predisposing factor is a history of previous scar-related VT. Some studies are ongoing to demonstrate that surgical ablation at the time of LVAD implant may decrease the occurrence of VT.^50–52^

In our centre, we implanted 722 LVAD in 656 patients. Catheter ablation of recurrent VT on LVAd was performed in 38 subjects (5.8% of all LVAD patients). In 12 cases, CA was accomplished in the first 30 days after LVAD implant (1.8%). This may not reflect the total number of electrical storms after implantation since some of them were managed by antiarrhythmic drugs, and only recurrent ones were ablated. Interestingly, 64 patients (9.8%) had CA prior LVAD implantation. Catheter ablation on LVAD is well tolerated and quite successful in abolishing VT. We contributed to an international collaboration group on this topic.^53^ Similar data were presented by others.^54^

As a bail-out strategy in high-risk patients who are not candidates for LVAD implant or heart transplant, STAR could be considered instead of CA supported by PMCS. In patients with destination LVAD, STAR could be considered a valuable palliative antiarrhythmic alternative with compassionate use as a last-resort strategy.^55,56^

In our opinion, PMCS may have some marginal role in enabling mapping during VT in the less-defined substrate, especially in patients with less advanced heart failure.

## Multidisciplinary approach

The above complexities of management of VA in patients with structural heart disease advocate for a multidisciplinary strategy, especially for patients with VT storm and advanced heart failure. This approach involves collaboration among specialists in acute cardiology, cardiac electrophysiologists, heart failure physicians, and cardiothoracic surgeons. This collective expertise aims to provide comprehensive care tailored to the complex needs of these patients. Some groups use such multidisciplinary management pathway based on a high PAINESD score.^57^ In our acute cardiology unit, we always attempt to evaluate clinical status, optimize haemodynamics, often use ganglion stellate blockade to suppress VA and provide the time window for subsequent decision whether to ablate or indicate the LVAD implant. We use the PAINESD score more like a predictor of mortality, and its value may influence our decision on advanced heart failure therapies rather than the use of PMCS for CA of VA.

## Conclusions

There is no clear-cut evidence supporting the broader use of PMCS in CA of VT in structural heart disease. Even in patients with severe left ventricular dysfunction and high PAINESD score, the substrate modification can be performed without the need for general anaesthesia or a risk of haemodynamic decompensation. In end-stage heart failure associated with recurrent VTs, implantation of LVAD (or PMCS with the transition to LVAD) might be the preferred strategy instead of CA on PMCS. In patients who are not potential candidates for these treatment options, STAR could be considered as a bail-out therapy for recurrent VTs. Alternatively, it may serve as a palliative therapy in patients with destination LVAD. These approaches should be studied in prospective trials. However, to organize a randomized trial on this topic would be very difficult for many reasons, including ethics.

## Data Availability

The data underlying this article are available in the article.

## References

[1] Mallidi J, Nadkarni GN, Berger RD, Calkins H, Nazarian S. Meta-analysis of catheter ablation as an adjunct to medical therapy for treatment of ventricular tachycardia in patients with structural heart disease. Heart Rhythm 2011;8:503–10. 21147263 10.1016/j.hrthm.2010.12.015 PMC3065522

[2] Aldhoon B, Wichterle D, Peichl P, Cihak R, Kautzner J. Outcomes of ventricular tachycardia ablation in patients with structural heart disease: the impact of electrical storm. PLoS One 2017;12:e0171830. 28187168 10.1371/journal.pone.0171830 PMC5302378

[3] Da Silva GL, Nunes-Ferreira A, Cortez-Dias N, de Sousa J, Pinto FJ, Caldeira D. Radiofrequency catheter ablation of ventricular tachycardia in ischemic heart disease in light of current practice: a systematic review and meta-analysis of randomized controlled trials. J Interv Card Electrophysiol 2020;59:603–16. 32948937 10.1007/s10840-020-00870-3

[4] Garcia JG, Arya A, Dinov B, Bollmann A, Ter Bekke RMA, Vernooy K et al Impact of repeat ablation of ventricular tachycardia in patients with structural heart disease. Europace 2023;26:euad367. 38127308 10.1093/europace/euad367 PMC10755192

[5] Mariani S, Napp LC, Lo Coco V, Delnoij TSR, Luermans JGLM, ter Bekke RMA et al Prophylactic mechanical support for protected ventricular tachycardia ablation. Int J Cardiol 2020;308:42–9. 32229050 10.1016/j.ijcard.2020.03.045

[6] Zanobini M, Rossi F, Bertera A, Sandano S, Costa C, Fabrizi R et al Cardiopulmonary support during electrophysiological procedures for ventricular tachycardias not haemodynamically tolerated. Perfusion 2003;18:79–82. 12868784 10.1191/0267659103pf651oa

[7] Carbucicchio C, Della Bella P, Fassini G, Trevisi N, Riva S, Giraldi F et al Percutaneous cardiopulmonary support for catheter ablation of unstable ventricular arrhythmias in high-risk patients. Herz 2009;34:545–52. 20091254 10.1007/s00059-009-3289-3

[8] Miller MA, Dukkipati SR, Mittnacht A, Chinitz JS, Belliveau L, Koruth JS et al Activation and entrainment mapping of hemodynamically unstable ventricular tachycardia using a percutaneous left ventricular assist device. J Am Coll CArdiol 2011;58:1363–71. 21920266 10.1016/j.jacc.2011.06.022

[9] Bunch RJ, Darby A, May HT, Ragosta M, Lim DS, Taylor AM et al Efficacy and safety of ventricular tachycardia ablation with mechanical circulatory support compared with substrate-based ablation techniques. Europace 2012;14:709–14. 22080473 10.1093/europace/eur347 PMC3598428

[10] Miller MA, Dukkipati SR, Chinitz JS, Koruth JS, Mittnacht AJ, Napolitano C et al Percutaneous hemodynamic support with Impella 2.5 during scar-related ventricular tachycardia ablation (PERMIT 1). Circ Arrhythm Electrophysiol 2013;6:151–6. 23255277 10.1161/CIRCEP.112.975888

[11] Lu F, Eckman PM, Liao KK, Apostolido I, John R, Chen T et al Catheter ablation of hemodynamically unstable ventricular tachycardia with mechanical circulatory support. Int J Cardiol 2013;168:3859–65. 23863501 10.1016/j.ijcard.2013.06.035

[12] Aryana A, O’Neill G, Gregory D, Scotti D, Bailey S, Brunton S et al Procedural and clinical outcomes after catheter ablation of unstable ventricular tachycardia supported by a percutaneous left ventricular assist device. Heart Rhythm 2014;11:1122–30. 24732372 10.1016/j.hrthm.2014.04.018

[13] Baratto F, Pappalardo F, Oloriz T, Bisceglia C, Vergara P, Silberbauer J et al Extracorporeal membrane oxygenation for hemodynamic support of ventricular tachycardia ablation. Arrhythm Electrophysiol 2016;9:e004492. 10.1161/CIRCEP.116.004492 27932426

[14] Turagam MK, Vuddanda V, Atkins D, Santangeli P, Frankel DS, Tung R et al Hemodynamic support in ventricular tachycardia ablation: an international VT ablation center collaborative group study. JACC Clin Electrophysiol 2017;26:1534–43. 10.1016/j.jacep.2017.07.005 29759835

[15] Mathuria N, Wu G, Rojas-Delgado F, Shuraih M, Razavi M, Civitello A et al Outcomes of pre-emptive and rescue use of left ventricular assist device in patients with structural heart disease undergoing catheter ablation of ventricular tachycardia. J Interv Card Electrophysiol 2017;48:27–34. 27497847 10.1007/s10840-016-0168-8

[16] Kusa S, Miller MA, Whang W, Enomoto Y, Panizo JG, Iwasawa J et al Outcome of ventricular tachycardia ablation using percutaneous left ventricular assist devices. Circ Arrhyt Electrophysiol 2017;10:e004717. 10.1161/CIRCEP.116.004717 28576780

[17] Muser D, Liang JJ, Castro SA, Hayashi T, Enriquez A, Troutman GS et al Outcomes with prophylactic use of percutaneous left ventricular assist devices in high-risk patients undergoing catheter ablation of scar-related ventricular tachycardia: a propensity-score matched analysis. Heart Rhythm 2018;15:1500–6. 29753944 10.1016/j.hrthm.2018.04.028

[18] Enriquez A, Liang J, Gentile J, Schaller R, Supple G, Frankel DS et al Outcomes of rescue cardiopulmonary support for periprocedural hemodynamic decompensation in patients undergoing catheter ablation of electrical storm. Heart Rhythm 2018;15:75–80. 28917560 10.1016/j.hrthm.2017.09.005

[19] Di Monaco A, Quadrini F, Troisy F, Vitulano N, Caruso R, Duni N et al Cardiopulmonary support in patients undergoing catheter ablation of poorly tolerated ventricular arrhythmias and electrical storm. J Cardiovasc Electrophysiol 2019;30:1281–6. 31111583 10.1111/jce.13995

[20] Neuzner J, Dietze T, PAliege R, Gradaus R. Effectiveness of a percutaneous left ventricular assist device in preventing acute hemodynamic decompensation during catheter ablation of ventricular tachycardia in advance heart failure patients: a retrospective single-center analysis. J Cardiac Electrophysiol 2019;30:2864–8. 10.1111/jce.14199 31549434

[21] Ballout JA, Wazni OM, Tarakji KG, Saliba WI, Kanj M, Diab M et al Catheter ablation in patients with cardiogenic shock and refractory ventricular tachycardia. Circ Arrhythm Electrophysiol 2020;13:e007669. 32281407 10.1161/CIRCEP.119.007669 PMC7285871

[22] Grimaldi M, Marino MM, Vitulano N, Quadrini F, Troisi F, Caporusso N et al Cardiopulmonary support during catheter ablation of ventricular arrhythmias with hemodynamic instability: the role of inducibility. Front Cardiovasc Med 2021;8:747858. 34746263 10.3389/fcvm.2021.747858 PMC8563579

[23] Chen Q, Pollet M, Mehta A, Wang S, Dean J, Parenti J et al Delayed removal of a percutaneous left ventricular assist device for patients undergoing catheter ablation of ventricular tachycardia is associated with increased 90-day mortality. J Interv Card Electrophysiol 2021;62:49–56. 32949304 10.1007/s10840-020-00875-y

[24] Friedman PA, Munger TM, Torres N, Rihal C. Percutaneous endocardial and epicardial ablation of hypotensive ventricular tachycardia with percutaneous left ventricular assist in the electrophysiology laboratory. J Cardiovasc Electrophysiol 2007;18:106–9. 17229307 10.1111/j.1540-8167.2006.00619.x

[25] Aryana A, d’Avila A, Cool CL, Miller MA, Garcia FC, Supple GE et al Outcomes of catheter ablation of ventricular tachycardia with mechanical hemodynamic support: an analysis of the Medicare database. J Cardiovasc Electrophysiol 2017;28:1295–302. 28800178 10.1111/jce.13312

[26] Santangeli P, Rame JE, Birati EY, Marchlinski FE. Management of ventricular arrhythmias in patients with advanced heart failure. J Am Coll Cardiol 2017;69:1842–960. 28385314 10.1016/j.jacc.2017.01.047

[27] Turagam MK, Vuddanda V, Koerber S, Garg J, Yarlagadda B, Dar T et al Percutaneous ventricular assist device in ventricular tachycardia ablation: a systematic review and meta-analysis. J Interv Cardiac Electrophysiol 2019;55:197–205. 10.1007/s10840-018-0477-1 30377926

[28] Mariani S, Napp JC, Kraaier K, Li T, Bounader K, Hanke JS et al Prophylactic mechanical circulatory support for protected ventricular tachycardia ablation: a meta-analysis of the literature. Artif Organs 2021;45:987–97. 33616221 10.1111/aor.13945

[29] Luni FK, Zungsontiporn N, Farid T, Malik SA, Khan S, Daniels J et al Percutaneous left ventricular assist device support during ablation of ventricular tachycardia: a meta-analysis of current evidence. J Cardiovasc Electrophysiol 2019;30:886–95. 30847997 10.1111/jce.13907

[30] Stojadinovic P, Wichterle D, Peichl P, ČIhák R, Aldhoon B, Borišincová E et al Periprocedural acute hemodynamic decompensation during substrate-based ablation of scar-related ventricular tachycardia: a rare and unpredictable event. Europace 2024;26:euae145. 38864730 10.1093/europace/euae145 PMC11167661

[31] Cuculich PS, Schill MR, Kashani R, Mutic S, Lang A, Cooper D et al Noninvasive cardiac radiation for ablation of ventricular tachycardia. N Engl J Med 2017;377:2325–36. 29236642 10.1056/NEJMoa1613773 PMC5764179

[32] Neuwirth R, Cvek J, Knybel L, Jiravsky O, Molenda L, Kodaj M et al Stereotactic radiosurgery for ablation of ventricular tachycardia. Europace 2019;21:1088–195. 31121018 10.1093/europace/euz133

[33] Jumeau R, Ozsahin M, Schwitter J, Vallet V, Duclos F, Zeverino M et al Rescue procedure for an electrical storm using robotic non-invasive cardiac radio-ablation. Radiother Oncol 2018;128:189–91. 29753550 10.1016/j.radonc.2018.04.025

[34] Ninni S, Gallot-Lavallée T, Klein C, Longere B, Brigadeau F, Potelle C et al Stereotactic radioablation for ventricular tachycardia in the setting of electrical storm. Circ Arrhythm Electrophysiol 2022;15:e010955. 36074658 10.1161/CIRCEP.122.010955

[35] Wight J, Bigham T, Schwartz A, ZAhid AT, Bhatia N, Kiani S et al Long term follow-up of stereotactic body radiation therapy for refractory ventricular tachycardia in advanced heart failure patients. Front Cardiovasc Med 2022;9:849113. 35571173 10.3389/fcvm.2022.849113 PMC9098944

[36] Hašková J, Wichterle D, Kautzner J, Šramko M, Peichl P, Jiravský O et al Efficacy and safety of stereotactic radiotherapy in patients with recurrent ventricular tachycardias: the Czech experience. JACC Clin Electrophysiol 2024;10:654–66. 38385912 10.1016/j.jacep.2023.12.002

[37] Benali K, Higgins K, Quivrin M, Bessieres I, WIght JA, Gupta D et al Cardiac stereotactic radiation therapy for refractory ventricular arrhythmias in patients with left ventricular assist device. JACC Clin Electrophysiol 2023;9:707–9. 37225312 10.1016/j.jacep.2022.12.006

[38] Benali K, LLoyd MS, Petrosyan A, Rigal L, Quivrin M, Bessieres I et al Cardiac stereotactic radiation therapy for refractory ventricular arrhythmias in patients with left ventricular assist devices. J Cardiovasc Electrophysiol 2024;35:206–13. 38018417 10.1111/jce.16139

[39] Kumar S, Baldinger SH, Romero J, Fujii A, Mahida SN, Tedrow UB et al Substrate-based ablation versus ablation guided by activation and entrainment mapping for ventricular tachycardia: a systematic review and meta-analysis. J Cardiovasc Electrophysiol 2016;27:1437–47. 27574120 10.1111/jce.13088

[40] Pourdjabbar A, Maze R, Hibbert B, Ruel M, Haddad H. Left ventricular assist device in the management of refractory electrical storm. Perfusion 2015;30:302–4. 25106413 10.1177/0267659114546033

[41] Tsai FC, Wang YC, Huang YK, Tseng SN, Wu MY, Chang Y-S et al Extracorporal life support to terminate refractory ventricular tachycardia. Crit Care Med 2007;35:1673–6. 17507822 10.1097/01.CCM.0000269030.57298.AF

[42] Kulick DM, Bolman ME III, Salerno CT, Bank AJ, Park SJ. Management of recurrent ventricular tachycardia with ventricular assist device placement. Ann Thorac Surg 1998;66:571–3. 9725415 10.1016/s0003-4975(98)00512-8

[43] Fasseas P, Kutalek SP, Samuels FL, Holmes EC, Samuels LE. Ventricular assist device support for management of sustained ventricular arrhythmias. Texas Heart Inst J 2002;29:33–6. PMC101266 11995847

[44] Efimova E, Fischer J, Bertagnolli L, Dinov B, Kircher S, Rolf S et al Predictors of ventricular arrhythmia after left ventricular assist device implantation: a large single-center observational study. Heart Rhythm 2017;14:1812–9. 28756099 10.1016/j.hrthm.2017.07.027

[45] Moss JD, Flatley EE, Beaser AD, Shin JH, Nayak HM, Upadhyay GA et al Characterization of ventricular tachycardia after left ventricular assist device implantation as destination therapy: a single-center ablation experience. J Am Coll Cardiol EP 2017;3:1412–24. 10.1016/j.jacep.2017.05.012 29759673

[46] Galand V, Flecher E, Auffret V, Pichard C, Boulé S, Vincentelli A et al Early ventricular arrhythmias after LVAD implantation is the strongest predictor of 30-day post-operative mortality. J Am Coll Cardiol 2019;5:944–54. 10.1016/j.jacep.2019.05.025 31439296

[47] Martins RP, Leclercq C, Bourenane H, Auffret V, Boulé S, Loobuyck V et al Incidence, predictors, and clinical impact of electrical storm in patients with left ventricular assist devices: new insights from the ASSIST-ICD study. Heart Rhythm 2019;16:1506–12. 31255846 10.1016/j.hrthm.2019.06.021

[48] Karikalan S, Tan MC, Zhang N, El-Masr H, Killu AM, DeSimone CV et al Electrical storm after left ventricular assist device (LVAD) implantation. J Cardiovasc Electrophysiol 2024;35:1196–202. 38590268 10.1111/jce.16275

[49] Rehorn MR, Black-Maier E, Loungani R, Sen S, Sun AY, Friedman DJ et al Electrical storm in patients with left ventricular assist devices: risk factors, incidence, and impact on survival. Heart Rhythm 2021;18:1263–71. 33839327 10.1016/j.hrthm.2021.03.047

[50] Patel M, Rojas F, Shabari FR, Simpson L, Cohn W, Frazier OH et al Safety and feasibility of open chest epicardial mapping and ablation of ventricular tachycardia during the period of left ventricular assist device implantation. J Cardiovasc Electrophysiol 2016;27:95–101. 26377813 10.1111/jce.12839

[51] Moss JD, Oesterle A, Raiman M, Flatley EE, Beaser AD, Jeevanandam V et al Feasibility and utility of intraoperative epicardial scar characterization during left ventricular assist device implantation. J Cardiovasc Electrophysiol 2019;30:183–92. 30516301 10.1111/jce.13803

[52] Huang DT, Gosev I, Wood KL, Vidula H, Stevenson W, Marchlinski F et al Design and characteristics of the prophylactic intra-operative ventricular arrhythmia ablation in high-risk LVAD candidates (PIVATAL) trial. Noninvasive Electrocardiol 2023;28:e13073. 10.1111/anec.13073 PMC10475893 37515396

[53] Sacher F, Reichlin T, Zado ES, Field ME, Viles-Gonzalez JF, Peichl P et al Characteristics of ventricular tachycardia ablation in patients with continuous flow left ventricular assist devices. Circ Arrhythm Electrophysiol 2015;8:592–7. 25870335 10.1161/CIRCEP.114.002394

[54] Anderson RD, Lee G, Virk S, Bennett RG, Hayward CS, Muthiah K et al Catheter ablation of ventricular tachycardia in patients with a ventricular assist device: a systematic review of procedural characteristics and outcomes. JACC Clin Electrophysiol 2019;5:39–51. 30678785 10.1016/j.jacep.2018.08.009

[55] Tseng YD, Gouwens NW, Lo SS, Halasz LM, Spady P, Mezheritsky IA et al Use of radiation therapy within the last year of life among cancer patients. Int J Radiat Oncol Biol Phys 2018;101:21–9. 29487025 10.1016/j.ijrobp.2018.01.056

[56] Spencer K, Parrish R, Barton R, Henry A. Palliative radiotherapy. BMJ 2018;360:k821. 29572337 10.1136/bmj.k821 PMC5865075

[57] Pothineni NVK, Enriques A, Kumareswaran R, Garcia F, Shah R, Wald J et al Outcomes of a PAINESD score-guided multidisciplinary management approach for patients with ventricular tachycardia storm and advanced heart failure: a pilot study. Heart Rhythm 2023;20:134–9. 36075533 10.1016/j.hrthm.2022.08.037

